# Correction to “Dynamic *In Vivo* Mapping of the Methylproteome Using a Chemoenzymatic Approach”

**DOI:** 10.1021/jacs.6c14984

**Published:** 2026-08-09

**Authors:** Jonathan Farhi, Benjamin Emenike, Richard S. Lee, Kirti Sad, Dorelle V. Fawwal, Christian M. Beusch, Robert B. Jones, Ashish K. Verma, Celina Y. Jones, Maryam Foroozani, Monica Reeves, Kiran K. Parwani, Pritha Bagchi, Roger B. Deal, David J. Katz, Anita H. Corbett, David E. Gordon, Monika Raj, Jennifer M. Spangle

At the time of initial peer review and publication, several data source files that represent Supporting Information were referenced in the main text but not included in the Supporting Information as indicated. These files are listed below and are attached to this submission. No existing files require changes or removal, only the addition of these below and attached files to the Supporting Information is required.

## Supplementary Material









## Notes

Mammalian Cell Analysis (XLSX)
GO_Analysis_Mammalian Cell (XLSX)
Mice Tissue Results (XLSX)
GO_Analysis_Mice_Tissue (XLSX)

