# Supplementary material for: Dynamic In Vivo Mapping of the Methylproteome Using a Chemoenzymatic Approach
Source: J Am Chem Soc. 2025 Feb 25;147(9):7214–30. doi: 10.1021/jacs.4c08175 (PMC11887452; doi:10.1021/jacs.4c08175)
Supplement: Supplementary file 1 — ja4c08175_si_001.pdf [file ja4c08175_si_001.pdf]

Dynamic *in vivo* mapping of the methylproteome using a chemoenzymatic approach

Jonathan Farhi[1,2,3,9], Benjamin Emenike[3,9], Richard S. Lee[1], Kirti Sad[1], Dorelle V. Fawwal[4], Christian M. Beusch[5], Robert B. Jones[1], Ashish K. Verma[3], Celina Y. Jones[7], Maryam Foroozani[7], Monica Reeves[8], Kiran K. Parwani[1,2], Pritha Bagchi[6], Roger B. Deal[7], David J. Katz[8], Anita H. Corbett[7], David E. Gordon[5], Monika Raj[3], Jennifer M. Spangle[1, *]

Extended Data

Supplementary Tables

Tables

Supplementary Table 1: CuAAC reaction conditions for experimental applications.

Application	CuSO4	THPTA	NaAsc	N3	DMSO	TX-100	Time
WB	1 mM	1.5 mM	3 mM	25 µM	None	None	30 m
IF	1 mM	1.5 mM	3 mM	25 µM	None	None	30 m
IHC	1 mM	1.5 mM	3 mM	25 µM	5%	0.2%	45 m
Biotin IP	1 mM	1.5 mM	3 mM	200 µM	None	None	1 h

Extended Figures

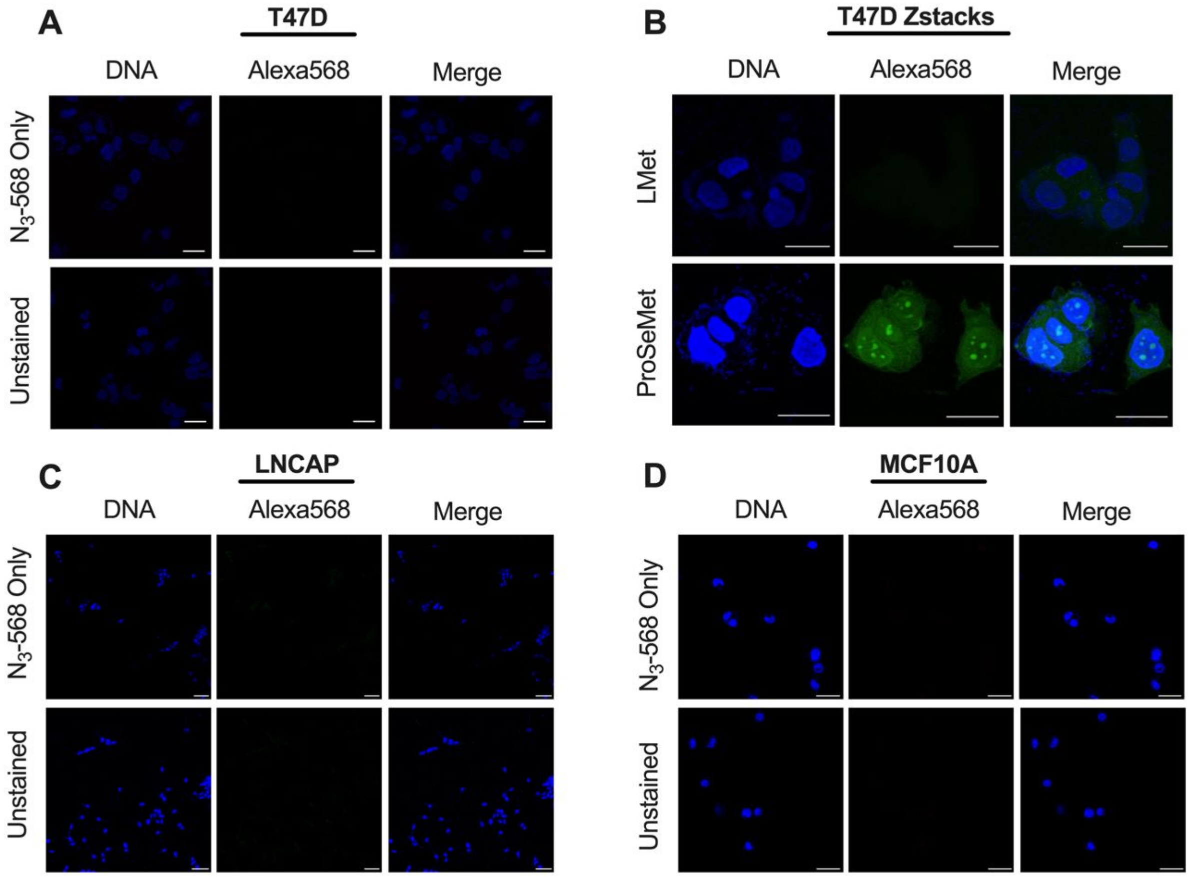

Supplementary Figure 1. IF controls for ProSeMet/L-Met labeling. IF controls for ProSeMet/L-Met labeling. T47D (**a**), LNCaP (**c**), and MCF10A (**d**) cells were treated with 100 µM ProSeMet or L-Met 16 h, fixed, permeabilized, and incubated with fluorescent picolyl azide alone or Hoechst stain alone. No nonspecific binding is observed in T47D, LNCaP, and MCF10A. Scale bar represents 25 µm (T47D, MCF10A) or 50 µM (LNCaP). **b.** T47D cells treated with ProSeMet or L-Met 16 h were fixed, permeabilized, and subjected to CuAAC to attach a fluorescent picolyl azide. Zstack image represents maximal intensity of each slice.

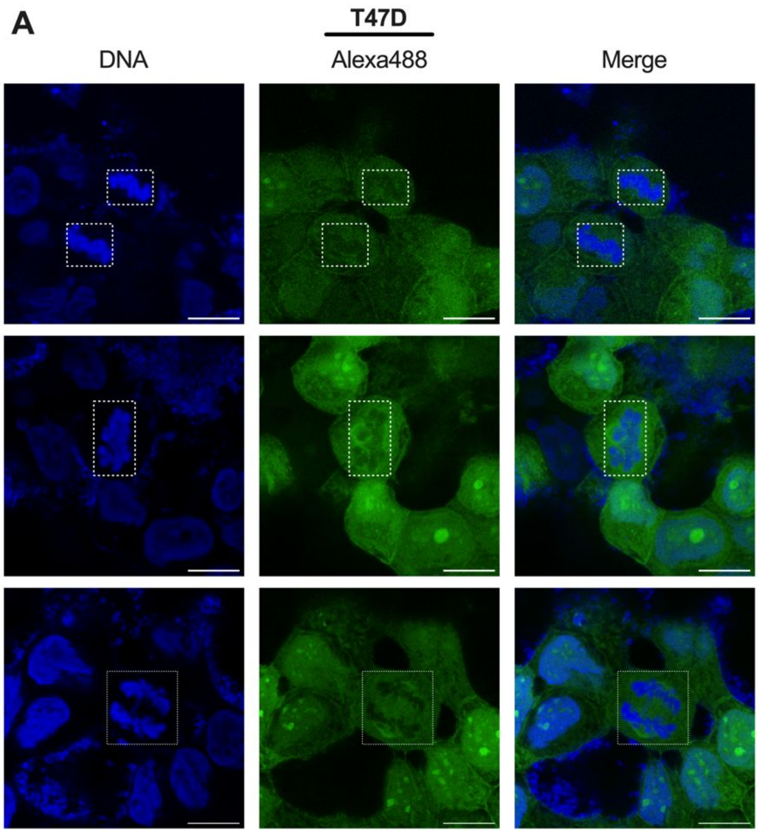

Supplementary Figure 2. ProSeMet does not label condensed DNA. T47D cells treated with 100 μM ProSeMet 16 h were fixed, permeabilized, and subjected to CuAAC to attach a fluorescent picolyl azide. **a**. Condensed DNA observed during cellular division (top: cytokinesis, middle: prophase, bottom: anaphase).

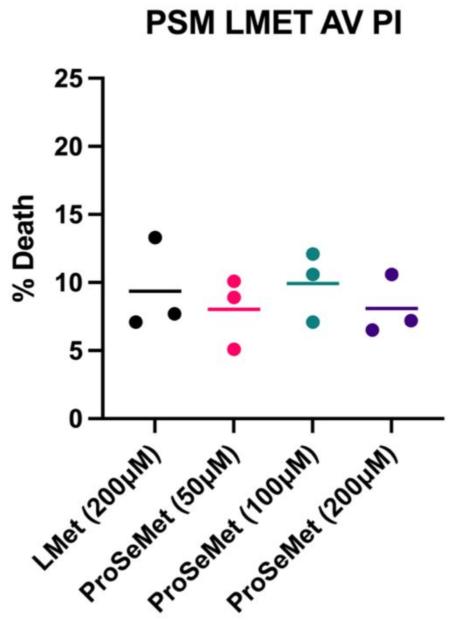

Supplementary Figure 3. ProSeMet treatment does not cause cell death. T47D cells treated with 50-200 μM ProSeMet for 24 h were stained with Annexin V/PI and analyzed by flow cytometry (*n=3*).

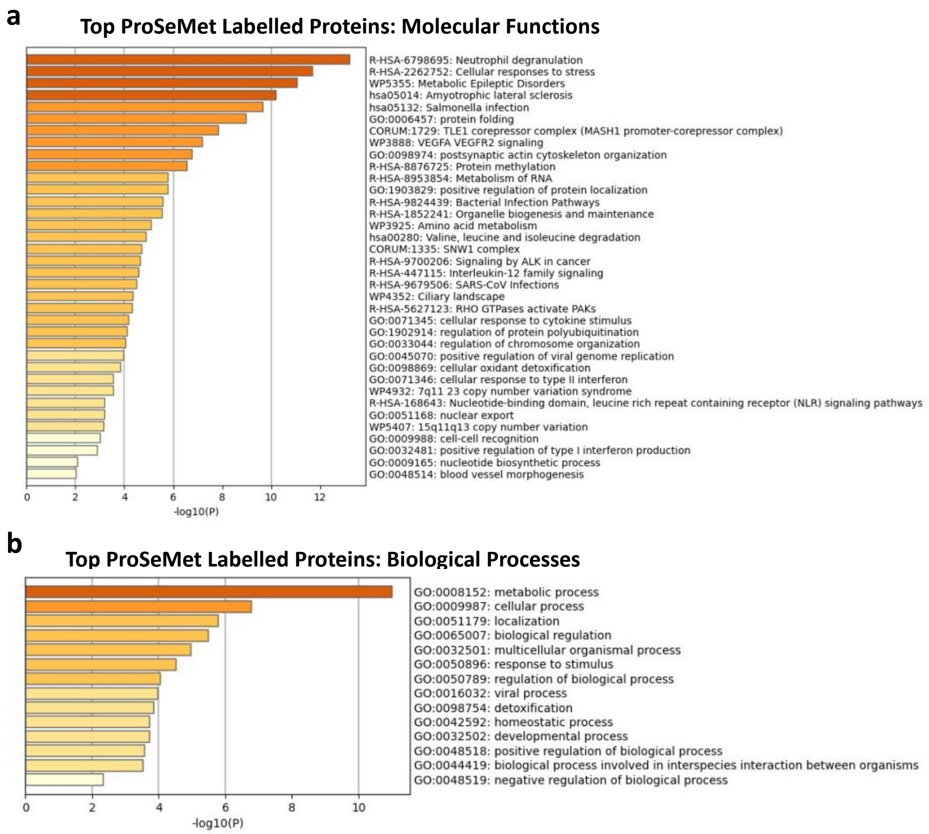

Supplementary Figure 4. Gene Ontology (GO) analysis of ProSeMet sites from unenriched LC-MS/MS in mammalian cells. (**a**) and (**b**). A gene list of propargylated proteins was utilized as input in metascape, with input and analysis species set to H. sapiens. Pathway and process enrichment analysis was carried out with the following ontology sources: KEGG Pathway, GO Biological Processes, Reactome Gene Sets, Canonical Pathways, CORUM, WikiPathways, and PANTHER Pathway. All genes in the human genome were used as the enrichment background. Terms with a p-value < 0.01, a minimum count of 3, and an enrichment factor > 1.5 were utilized. p-values were calculated based on the cumulative hypergeometric distribution, and q-values are calculated using the Benjamini-Hochberg procedure. The best term within a group is chosen as the group summary. ***Excel sheet containing analysis can be found in Supplementary data 2.***

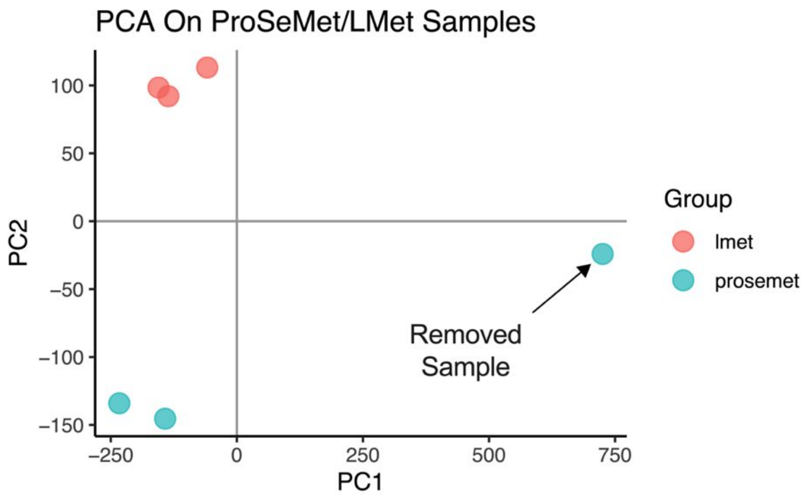

Supplementary Figure 5. PCA analysis of biological replicates submitted for LC-MS/MS analysis. PCA analysis of biological replicates submitted for LC-MS/MS analysis, generated via statistical software. ProSeMet sample 3 was removed prior to further analysis.

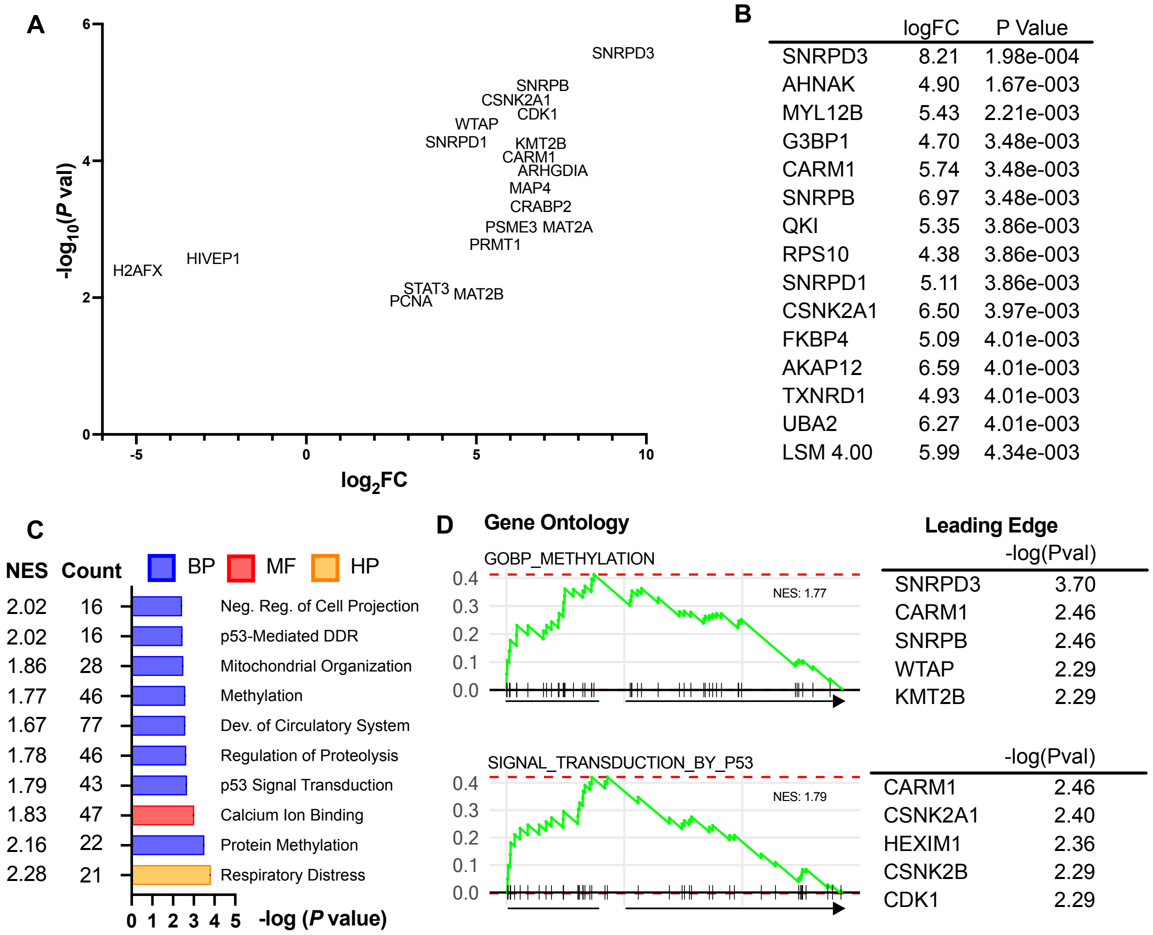

Supplementary Figure 6. Pseudomethylated proteins converge into predicted gene ontologies while extending the methylproteome. G401 cells treated with 100 µM ProSeMet or 100 µM L-Met 16 h were lysed, subjected to a click reaction to attach a biotin-conjugated picolyl azide, and enriched using magnetic streptavidin beads. Enriched proteins were digested with trypsin and analyzed via label-free LC-MS/MS. **a.** Volcano plot showing statistical significance vs $\log_2$-fold change (logFC) of proteins isolated from G401 cells treated with ProSeMet or L-Met. Data represents 3 biological replicates consisting of matched experiments. Statistical significance was calculated using a two-tailed unpaired *t*-test. **b**. Top 15 enriched proteins. Statistical significance represents scaled *P* values after differential expression analysis. **c**. Gene set enrichment analysis (GSEA) of ProSeMet enriched proteins using GO gene set. The top ten pathways are shown with corresponding number of proteins (count) and normalized enrichment score (NES). **d**. GSEA analysis of methylation (top) and p53-mediated signal transduction (bottom) gene sets. Top 5 enriched proteins by *P* value in the leading edge are shown to the right. For **c** and **d**, statistical significance was assessed using Fast Gene Set Enrichment Analysis (fgsea).

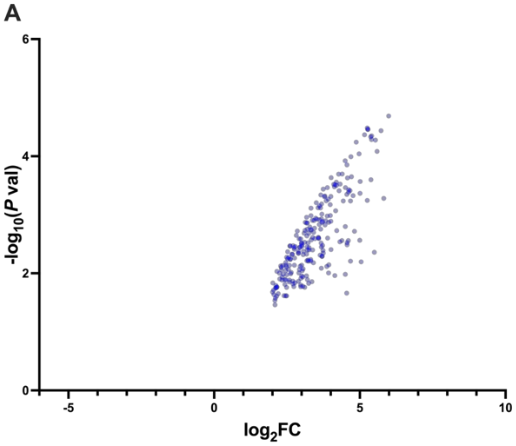

	logFC	logPValue
LSM 4.00	5.989810	4.686916
S100A1	5.256397	4.482155
CDK1	5.280067	4.460858
EPHA2	5.275312	4.454965
HMOX2	5.728186	4.437184
WTAP	5.163824	4.366803
IFITM3	5.406214	4.348426
ISG15	5.373568	4.331429
CARS	5.399502	4.284220
TNFRSF10B	5.533544	4.275322

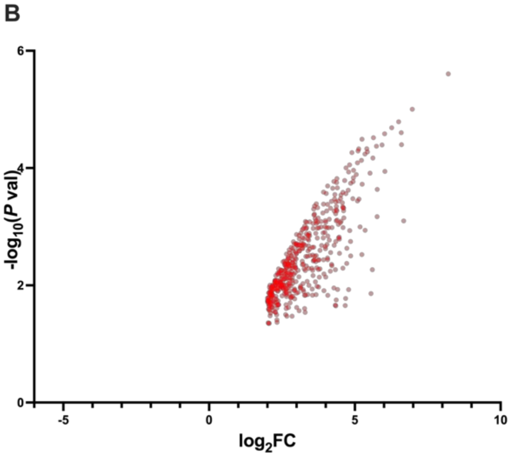

	logFC	logPValue
SNRPD3	8.206059	5.606909
SNRPB	6.973263	5.004295
CSNK2A1	6.502378	4.789590
UBA2	6.265811	4.686400
AKAP12	6.594047	4.603001
PABPN1	6.021634	4.585162
RPA2	5.640058	4.517992
HIGD2A	5.245443	4.492654
HEXIM1	6.604102	4.399010
KMT2B	5.933474	4.393224

Supplementary Figure 7. Analysis of ProSeMet-labeled proteome identified 707 proteins with uncharacterized methylation sites using PhosphoSitePlus database. Significantly enriched proteins identified *via* LC-MS/MS (p-value < 0.05, logFC > 2) were screened against the PhosphoSitePlus database of proteins with known methylation sites to identify (**a**) 221 proteins with uncharacterized methylation sites and (**b**) 486 proteins with previously identified methylation sites. Table to the right of each volcano plot indicates the top ten identified genes by p value.

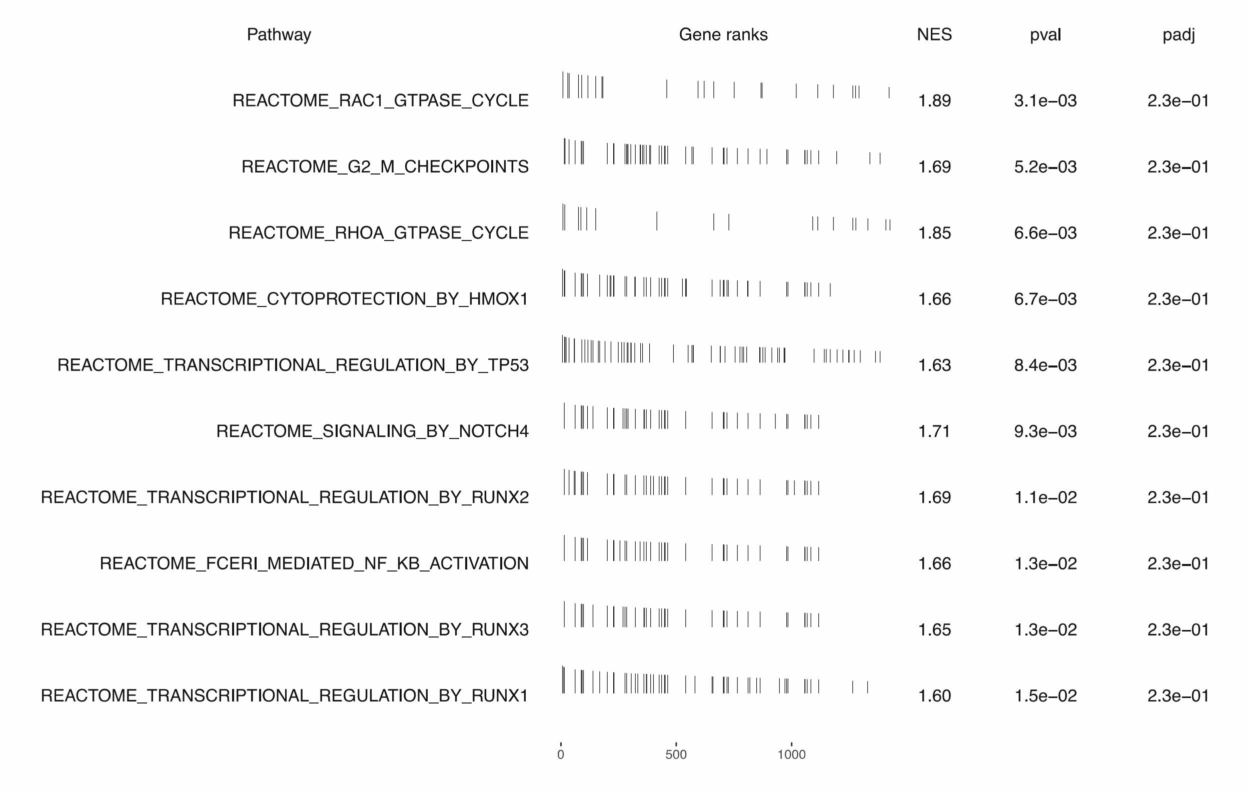

Supplementary Figure 8. GSEA analysis of ProSeMet enriched proteins using REACTOME molecular signatures database. GSEA analysis of ProSeMet enriched proteins using REACTOME gene set. The top ten pathways are shown with gene distribution based on rankings, normalized enrichment score (NES), *P* value, and adjusted *P* value (calculated via statistical software).

HSPA8: R469 (previously discovered)

Representative peptide spectra of modified peptides R469 propargylation (propargyl arginine site, +38 on arginine). Sequence: LTGIPPAPRGVPQIE, (+38.01570 Da on arginine).

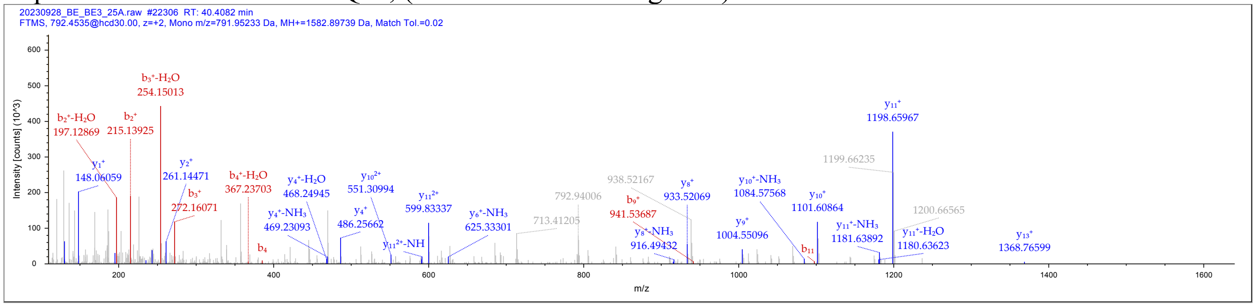

PGAM1: K222, K225, and K228 (novel)

Representative peptide spectra of modified peptides K222 propargylation (propargyl arginine site, +114.0471 on lysine). Sequence: LSEEAIMELNLPTGIPIVYELDK, (+114.0471 Da on lysine).

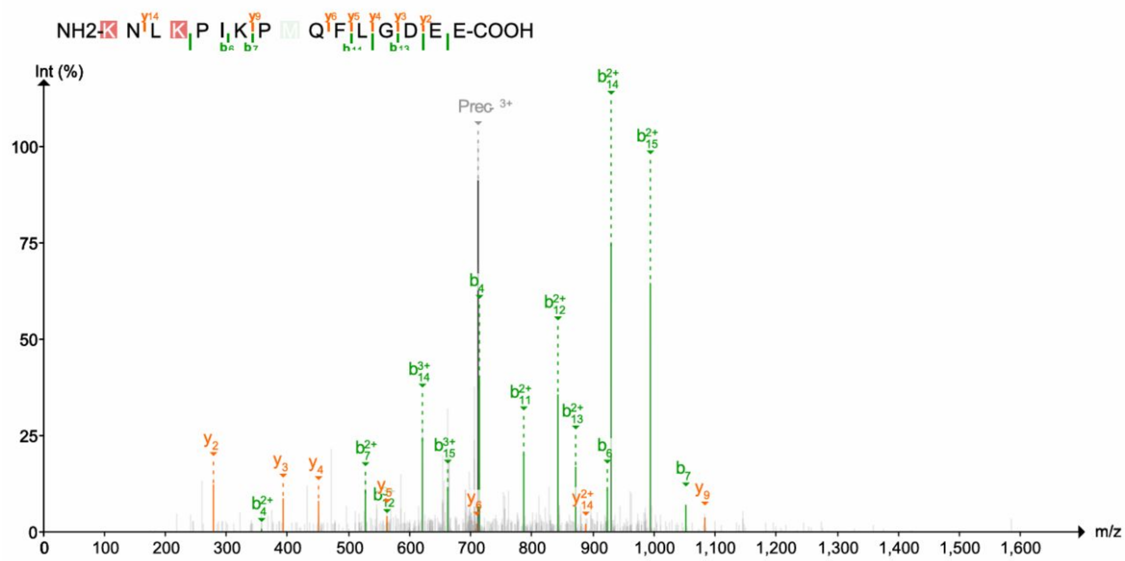

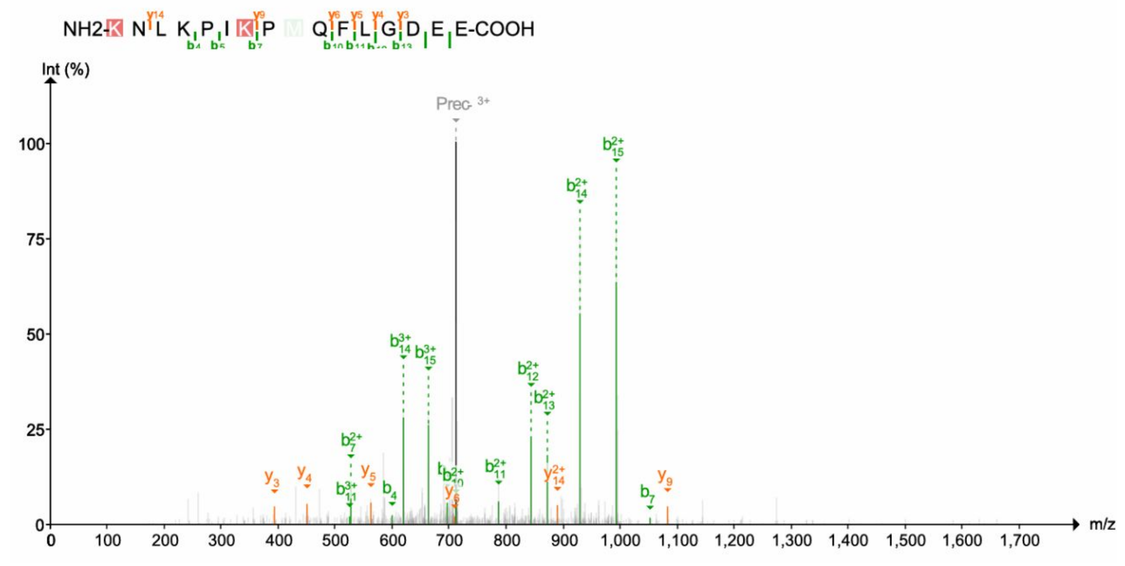

eEF1A1: K273 and K392 (novel)

Representative peptide spectra of modified peptides K273 propargylation (propargyl arginine site, +38.01570 Da on lysine).
Sequence: DGPKFLKSGDAAIVD, (+38.01570 Da on lysine, and +15.9949 Da on methionine).

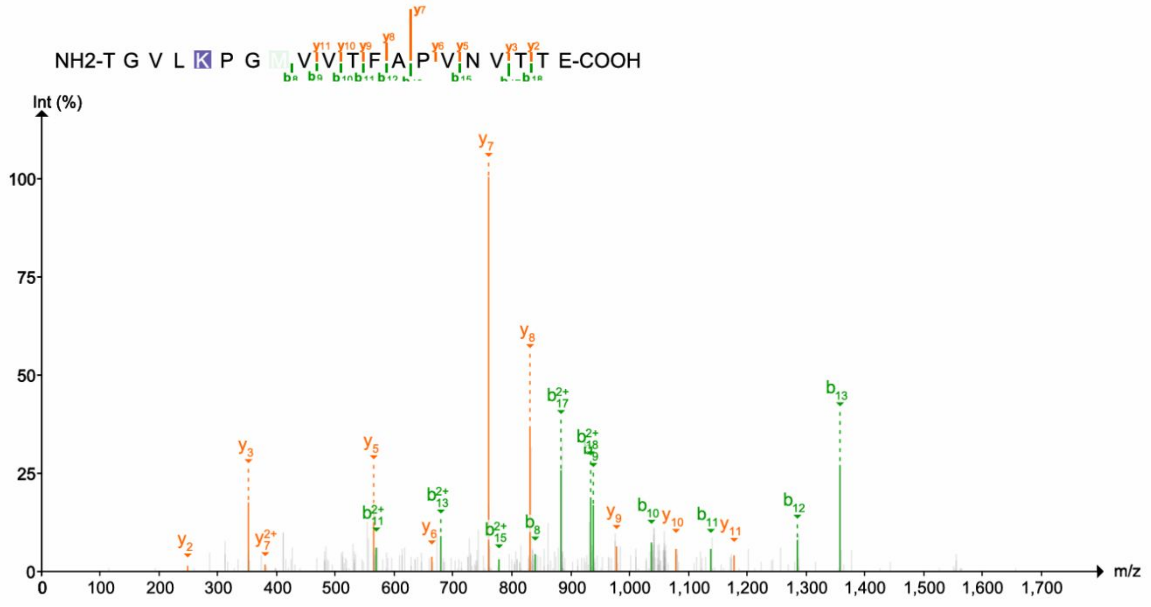

Representative peptide spectra of modified peptides K392 propargylation (propargyl arginine site, +38.01570 Da on lysine).
Sequence: NPKSLKSGDAAIVEMVPGKPMCVE, (+38.01570 Da on lysine, +15.9949 Da on methionine, and +57.0215 Da on cysteine).

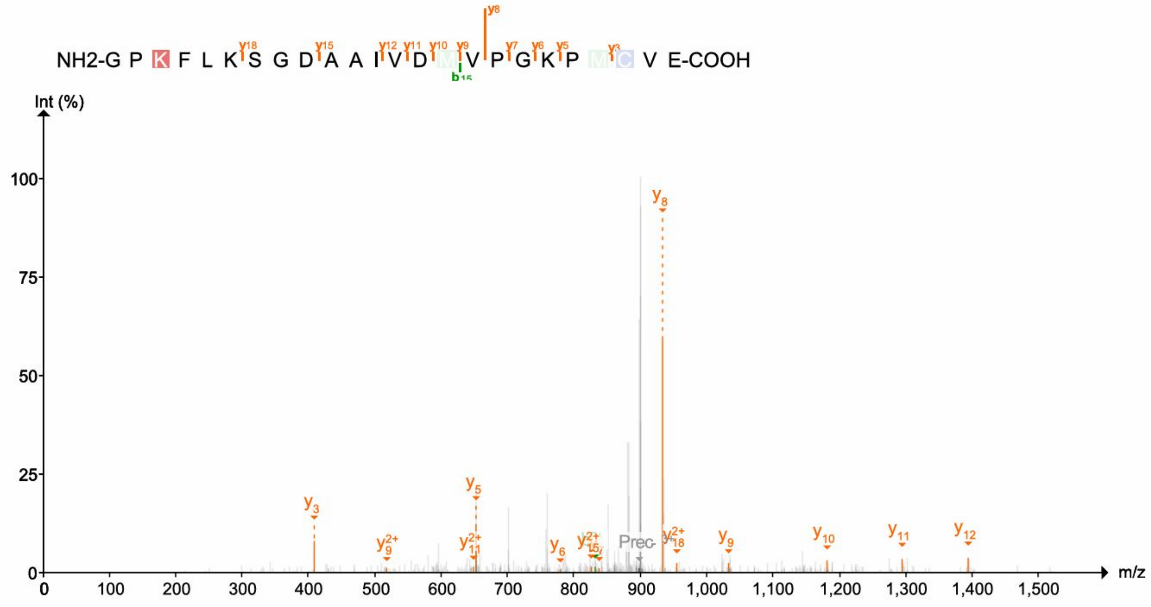

CALM1: R38 (novel)

Representative peptide spectra of modified peptides R38 propargylation (propargyl arginine site, +38 on arginine). Sequence: LGTVMRSLGQNPTEAE, (+38.01570 Da on arginine, and +15.9949 Da on methionine).

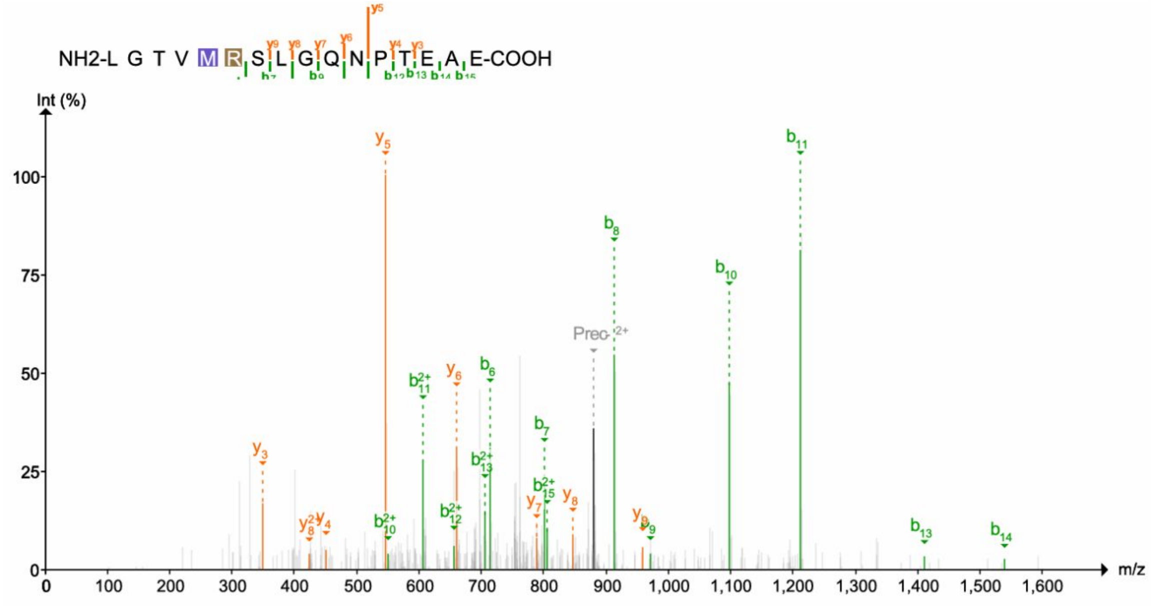

PGAM1: K222 validation through IP LC-MS/MS analysis.
Representative peptide spectra of modified peptides K222 propargylation (propargyl arginine site, +38 on lysine). Sequence: LSEEAIMELNLPTGIPIVYELDK, (+38.01570 Da on lysine).

MS1 spectra of modified peptide showing +2 and +3 charge state of peptide

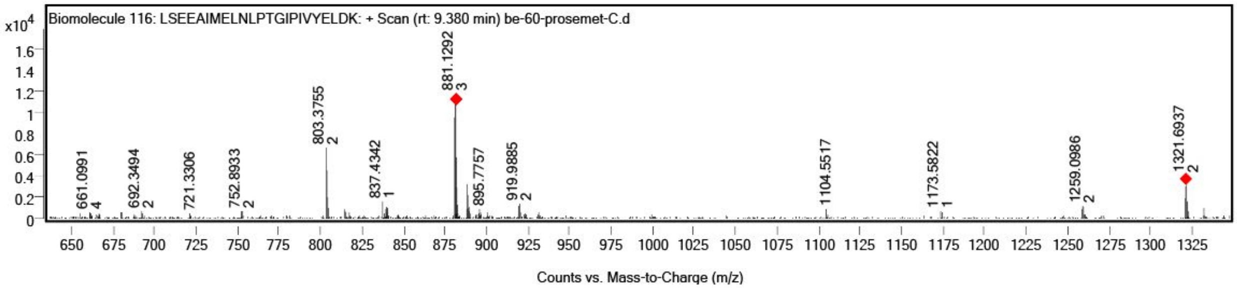

MS2 spectra of modified peptide showing y and b ions of peptide

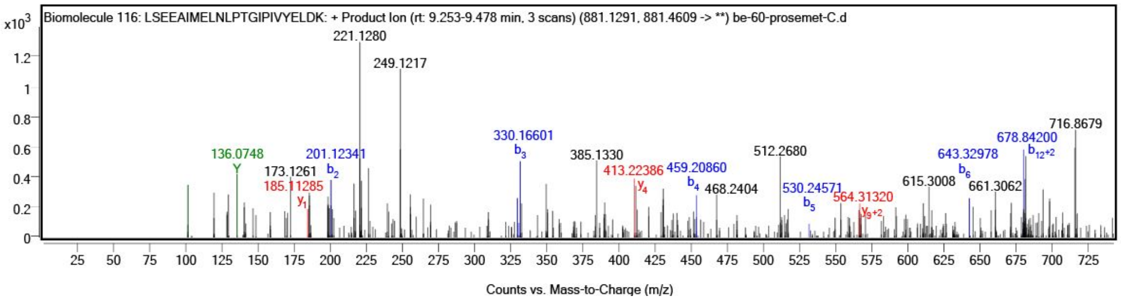

Fragment Ion Table, monoisotopic masses

Seq	#	B	Y	# (+1)
L	1	114.09139	2641.35757	23
S	2	201.12341	2528.27351	22
E	3	330.16601	2441.24148	21
E	4	459.20860	2312.19889	20
A	5	530.24571	2183.15630	19
I	6	643.32978	2112.11918	18
M	7	790.37026	1999.03512	17
E	8	919.41285	1851.99463	16
L	9	1032.49692	1722.95204	15
N	10	1146.53985	1609.86798	14
L	11	1259.62391	1495.82505	13
P	12	1356.67667	1382.74099	12
T	13	1457.72435	1285.68822	11
G	14	1514.74581	1184.64054	10
I	15	1627.82988	1127.61908	9
P	16	1724.88264	1014.53502	8
I	17	1837.96670	917.48225	7
V	18	1937.03512	804.39819	6
Y	19	2100.09845	705.32978	5
E	20	2229.14104	542.26645	4
L	21	2342.22510	413.22386	3
D	22	2457.25205	300.13979	2
K	23	2623.34701	185.11285	1

Mass/Charge Table

	Mass	
	Mono	Avg
(M)	2640.35030	2642.00828
$(M+H)^{+}$	2641.35757	2643.01556
$(M+2H)^{2+}$	1321.18245	1322.01144
$(M+3H)^{3+}$	881.12407	881.67673
$(M+4H)^{4+}$	661.09488	661.50938

Confirmation of ProSeMet as a methylation surrogate

eEF1A1 methylation site: K273 (novel)

Representative peptide spectra of modified peptide K273 methylation (monomethyl lysine site, +14.01570 Da on lysine). Sequence: TGVLKPGMVVTFAPVNVTTE, (+14.01570 Da on lysine).

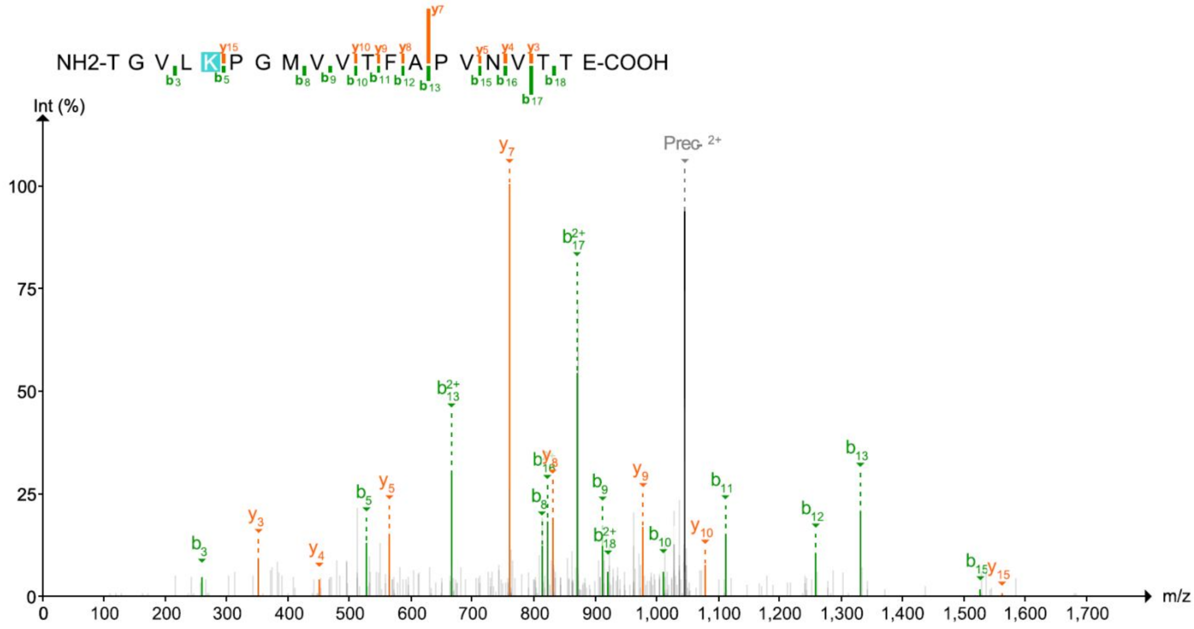

Representative peptide spectra of modified peptide K392 methylation (dimethyl lysine site, +28.0313 Da on lysine). Sequence: DGPkFLKSGDAAIVD, (+28.0313 Da on lysine).

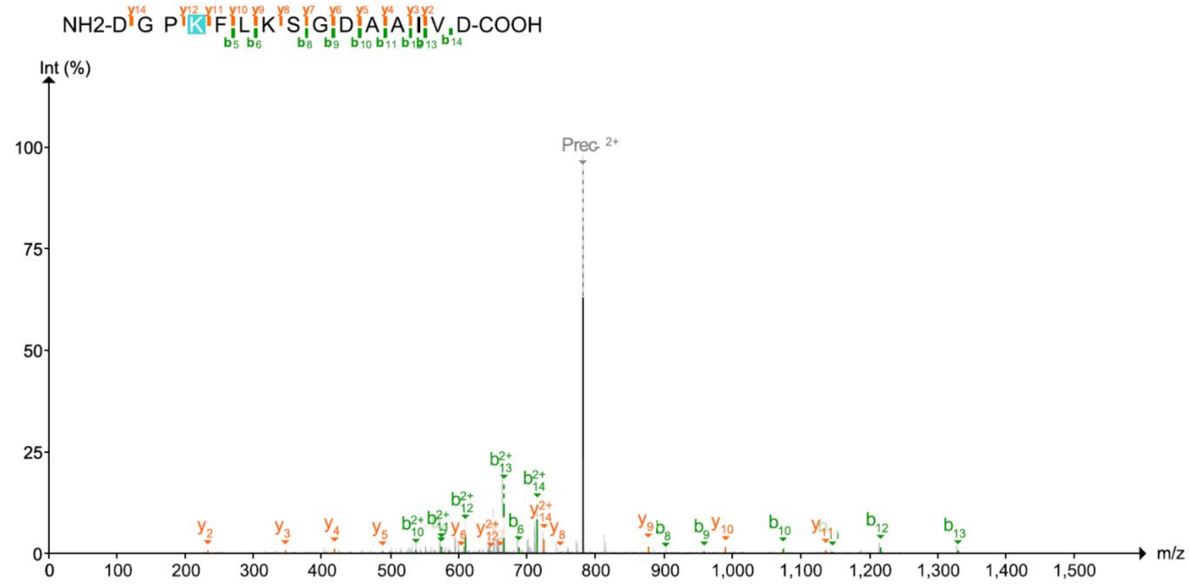

CALM1 methylation site: R38 (novel)

Representative peptide spectra of modified peptides R38 methylation (dimethyl arginine site, +28 on arginine). Sequence: LGTVMRSLGQNPTEAE, (+28.0313 Da on arginine).

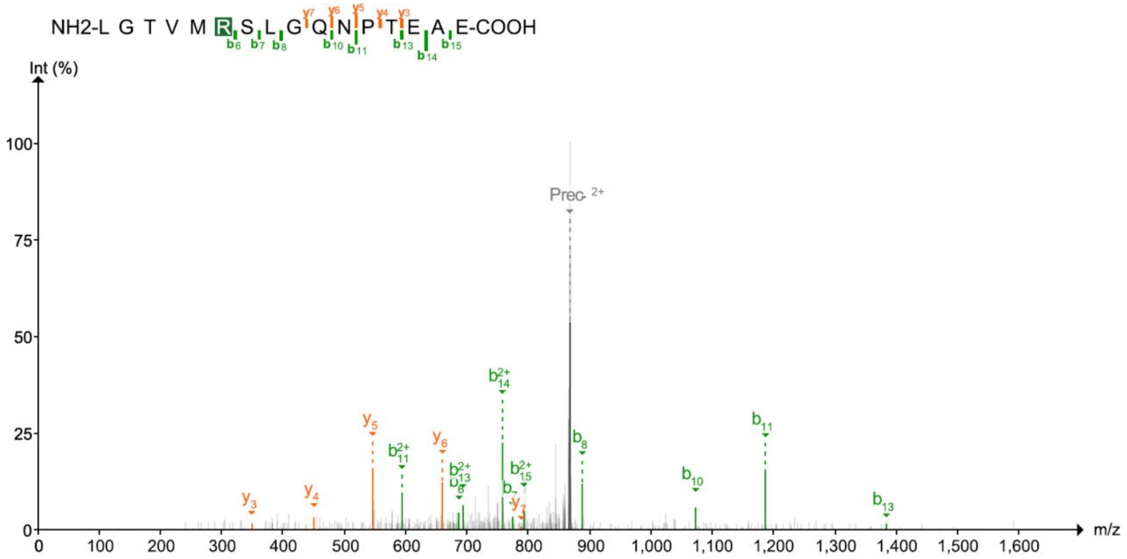

Supplementary Figure 9 (Pages 10-15). Representative spectra of previously discovered methylated HSPA8 R469 and novel propargylated and methylated protein substrates (PGAM1, eEF1A1, and CALM1).

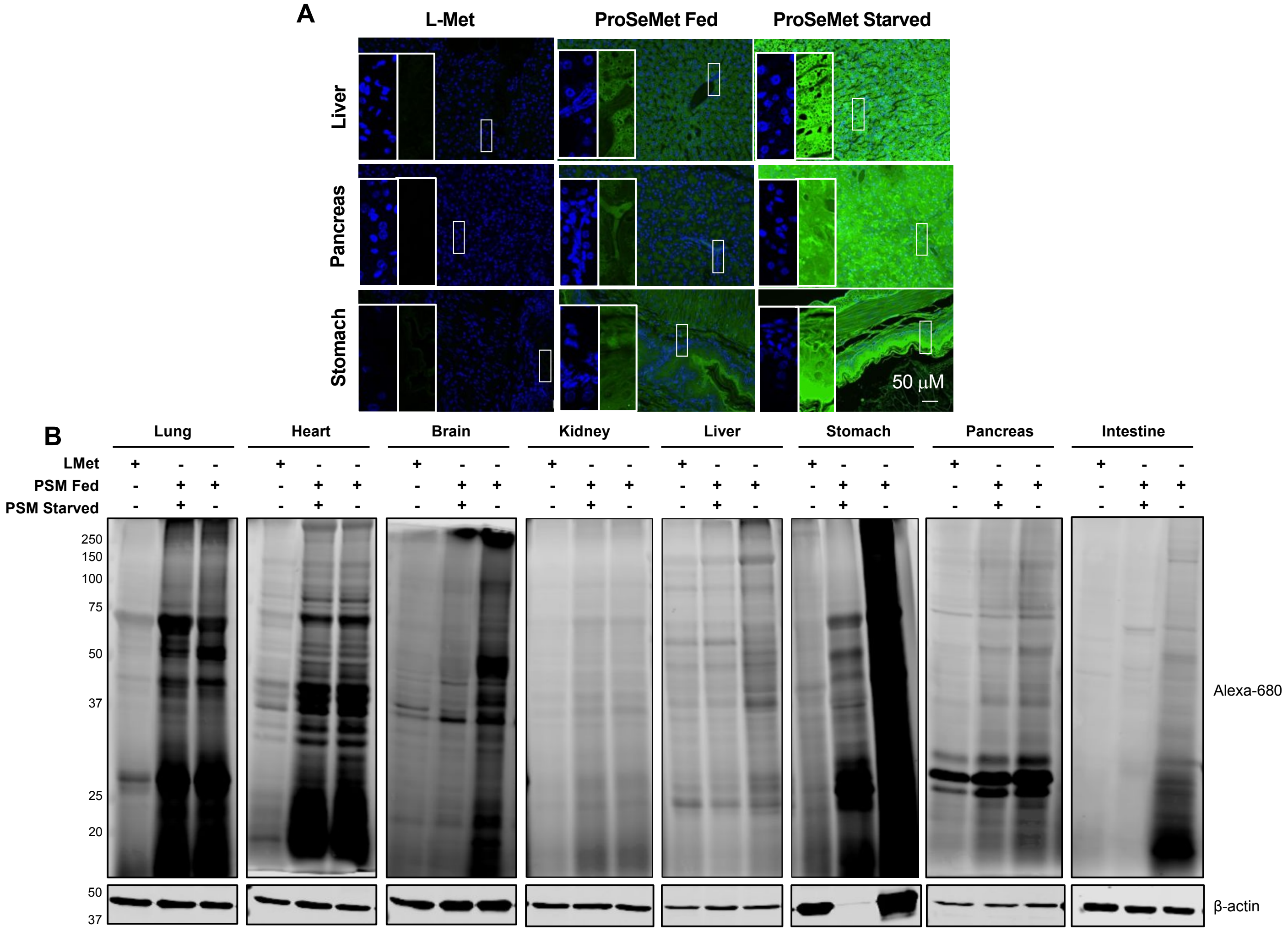

Supplementary Figure 10. In-tissue fluorescence and blot analysis of organs collected from mouse treated with ProSeMet or L-Met. Mice treated with ProSeMet or L-Met in the presence of food or starved were euthanized and organs were collected. **a.** Organs extracted from mice treated with 15 mg ProSeMet or equimolar L-Met via IP injection while being fed or starved for 12 h were formaldehyde fixed and paraffin embedded. Tissue sections were subjected to CuAAC to attach a fluorescent picolyl azide (568 nm, pseudocolored green) and counterstained with DAPI (blue). In-tissue fluorescence analysis of liver (top), pancreas (middle), and stomach (bottom), $n \geq 3$. **b.** Representative blot images to accompany the densitometry shown in Figure 5h, $n \geq 3$.

a **Top ProSeMet Labelled Proteins: Brain**

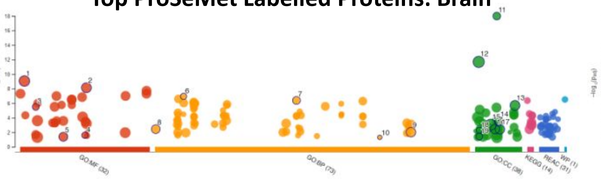

ID	Source	Term ID	Term Name	P_{adj} (query_1)
1	GO:MF	GO:0003824	catalytic activity	8.923×10^{-10}
2	GO:MF	GO:0043168	anion binding	7.488×10^{-9}
3	GO:MF	GO:0005200	structural constituent of cytoskeleton	3.202×10^{-6}
4	GO:MF	GO:0042288	MHC class I protein binding	2.588×10^{-2}
5	GO:MF	GO:0019901	protein kinase binding	4.160×10^{-2}
6	GO:BP	GO:0006734	NADH metabolic process	1.163×10^{-7}
7	GO:BP	GO:0046034	ATP metabolic process	4.167×10^{-7}
8	GO:BP	GO:0000226	microtubule cytoskeleton organization	3.685×10^{-3}
9	GO:BP	GO:1901566	organonitrogen compound biosynthetic process	9.718×10^{-3}
10	GO:BP	GO:0098735	positive regulation of the force of heart contraction	4.995×10^{-2}
11	GO:CC	GO:0043209	myelin sheath	1.036×10^{-18}
12	GO:CC	GO:0005737	cytoplasm	2.081×10^{-12}
13	GO:CC	GO:0120025	plasma membrane bounded cell projection	2.034×10^{-6}
14	GO:CC	GO:0044297	cell body	2.916×10^{-4}
15	GO:CC	GO:0032991	protein-containing complex	9.028×10^{-4}
16	GO:CC	GO:0036477	somatodendritic compartment	3.625×10^{-3}
17	GO:CC	GO:0045202	synapse	3.890×10^{-3}
18	GO:CC	GO:0005790	smooth endoplasmic reticulum	5.285×10^{-3}
19	GO:CC	GO:0005791	rough endoplasmic reticulum	3.885×10^{-2}

b **Top ProSeMet Labelled Proteins: Heart**

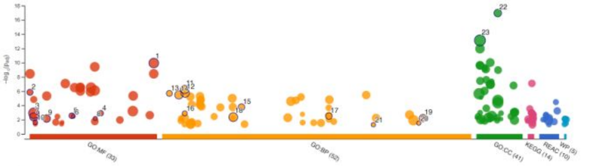

ID	Source	Term ID	Term Name ↑	P_{adj} (query_1)
6	GO:MF	GO:0030060	L-malate dehydrogenase activity	3.340×10^{-3}
10	GO:MF	GO:0003985	acetyl-CoA C-acetyltransferase activity	2.328×10^{-2}
7	GO:MF	GO:0003779	actin binding	3.928×10^{-3}
8	GO:MF	GO:0043532	angiostatin binding	6.672×10^{-3}
9	GO:MF	GO:0005516	calmodulin binding	6.838×10^{-3}
18	GO:BP	GO:0019752	carboxylic acid metabolic process	4.333×10^{-3}
3	GO:MF	GO:0003824	catalytic activity	1.198×10^{-3}
23	GO:CC	GO:0005737	cytoplasm	7.446×10^{-14}
11	GO:BP	GO:0006091	generation of precursor metabolites and energy	5.646×10^{-7}
1	GO:MF	GO:1901363	heterocyclic compound binding	1.119×10^{-10}
21	GO:BP	GO:0090131	mesenchyme migration	4.996×10^{-2}
2	GO:MF	GO:0000146	microfilament motor activity	1.416×10^{-6}
14	GO:BP	GO:0003012	muscle system process	3.315×10^{-6}
22	GO:CC	GO:0043209	myelin sheath	1.098×10^{-17}
15	GO:BP	GO:0030239	myofibril assembly	1.706×10^{-4}
20	GO:BP	GO:1902109	negative regulation of mitochondrial membrane p...	3.001×10^{-2}
16	GO:BP	GO:0006107	oxaloacetate metabolic process	1.277×10^{-3}
5	GO:MF	GO:0019825	oxygen binding	2.745×10^{-3}
19	GO:BP	GO:1902600	proton transmembrane transport	5.828×10^{-3}
4	GO:MF	GO:0046933	proton-transporting ATP synthase activity, rotation...	1.219×10^{-3}
12	GO:BP	GO:0006163	purine nucleotide metabolic process	1.827×10^{-6}
13	GO:BP	GO:0002026	regulation of the force of heart contraction	2.037×10^{-6}
17	GO:BP	GO:0055010	ventricular cardiac muscle tissue morphogenesis	3.041×10^{-3}

c **Top ProSeMet Labelled Proteins: Lung**

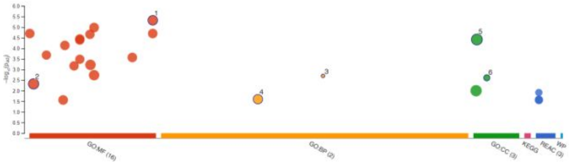

ID	Source	Term ID	Term Name	P_{adj} (query_1)
1	GO:MF	GO:1901363	heterocyclic compound binding	4.779×10^{-6}
2	GO:MF	GO:0003824	catalytic activity	4.747×10^{-3}
3	GO:BP	GO:0051659	maintenance of mitochondrion location	1.997×10^{-3}
4	GO:BP	GO:0033554	cellular response to stress	2.495×10^{-2}
5	GO:CC	GO:0005737	cytoplasm	3.834×10^{-5}
6	GO:CC	GO:0031672	A band	2.449×10^{-3}

Supplementary Figure 11. Gene Ontology (GO) analysis of ProSeMet sites from *in vivo* murine tissue. Gene lists of propargylated proteins from murine brain (**a**), heart (**b**), and lung (**c**) tissue-derived LC-MS/MS were utilized as input in metascape and gProfiler, with input and analysis species set to *H. sapiens*. Pathway and process enrichment analysis was carried out with the following ontology sources: KEGG Pathway, GO Biological Processes, Reactome Gene Sets, Canonical Pathways, CORUM, WikiPathways, and PANTHER Pathway. All genes in the *M. Musculus* genome were used as the enrichment background. Terms with a p-value < 0.05, a minimum count of 3, and an enrichment factor > 1.5 were utilized. p-values were calculated based on the cumulative hypergeometric distribution, and q-values are calculated using the Benjamini-Hochberg procedure. ***Excel sheet containing analysis can be found in Supplementary data 4.***

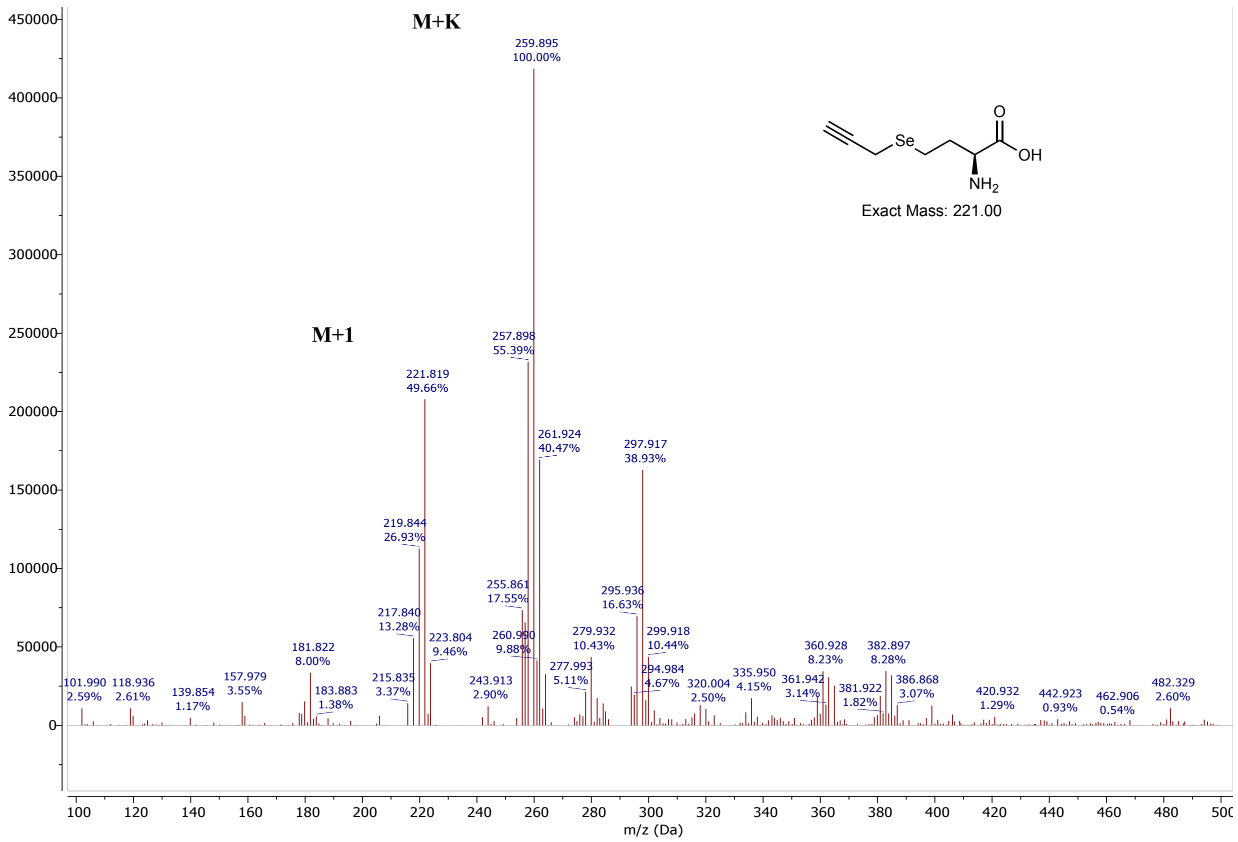

Supplementary Figure 12. ESI-MS of synthesized ProSeMet. Expected mass for $C_7H_{11}NO_2SeK^+$: 259.24 $[M + K]^+$; found: 259.815.

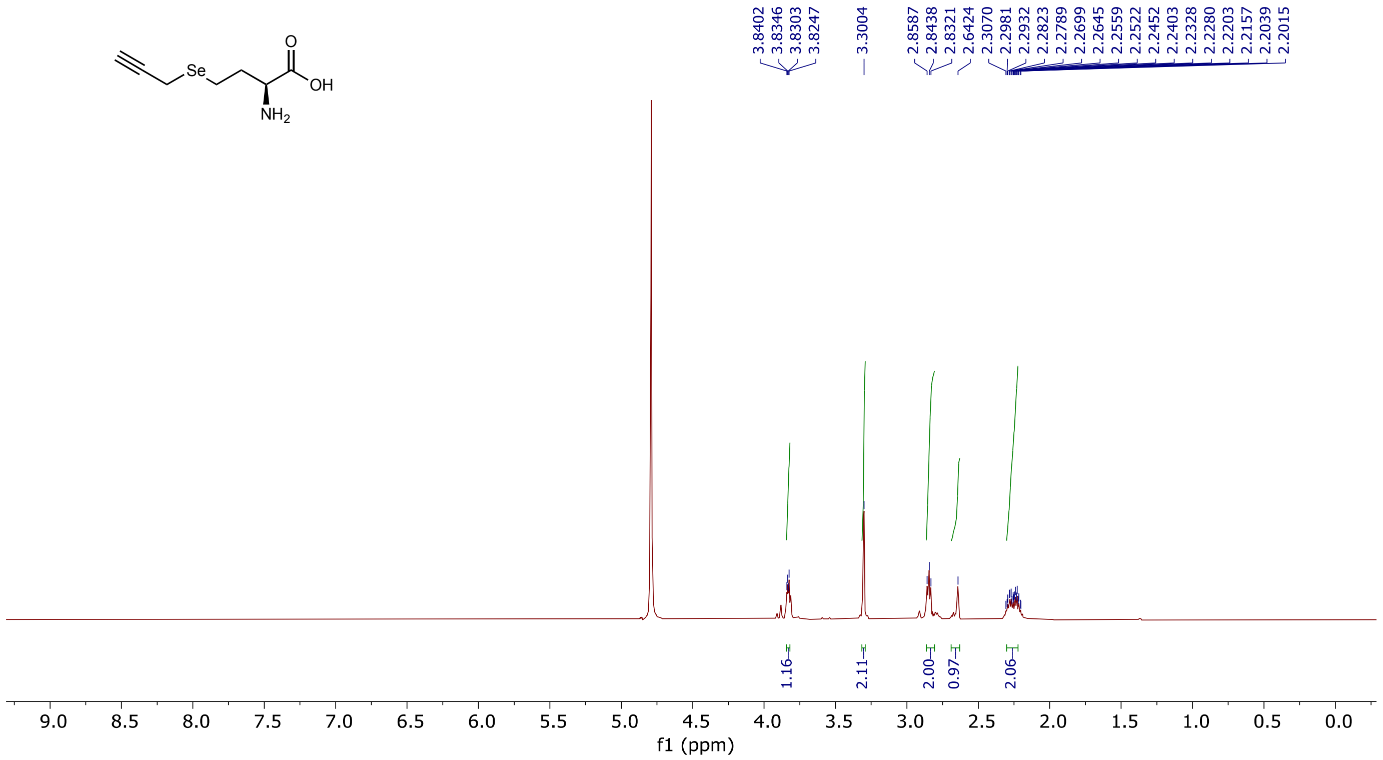

Supplementary Figure 13. ^{1}H NMR of synthesized ProSeMet. Purified ProSeMet was dissolved in dH2O and analyzed by ^{1}H NMR (600 MHz). ^{1}H NMR (600 MHz, D2O) δ 3.91-3.66 (m, 1H), 3.15 (s, 2H), 2.69 (dd, J = 9.1, 6.8 Hz, 2H), 2.49 (s, 1H), 2.14 – 2.07 (m, 2H).

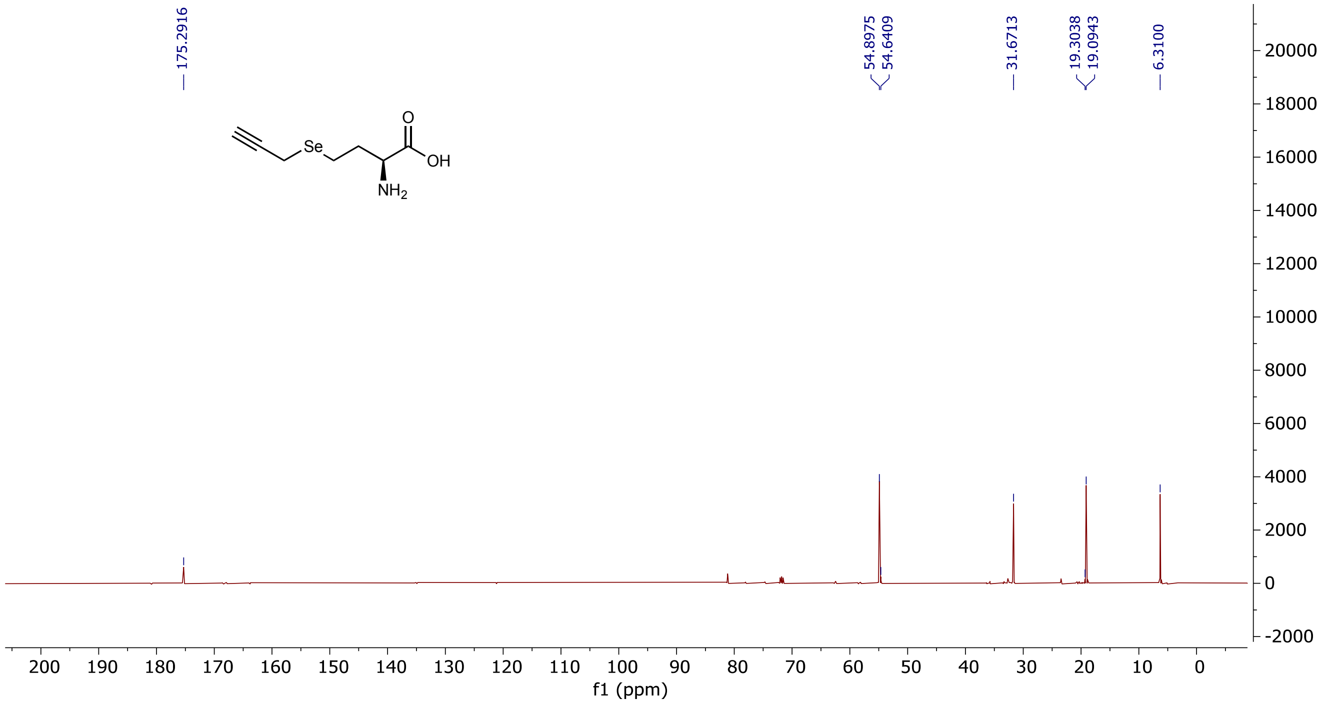

Supplementary Figure 14. ^{13}C NMR of synthesized ProSeMet. Purified ProSeMet was dissolved in dH2O and analyzed by ^{13}C NMR (151 MHz). ^{13}C NMR (151 MHz, D2O) δ 175.29, 54.90, 54.64, 31.67, 19.30, 19.09, 6.31.
